# Molecular thresholds of ITS2 and their implications for molecular evolution and species identification in seed plants

**DOI:** 10.1038/s41598-017-17695-2

**Published:** 2017-12-11

**Authors:** Ying Qin, Meihui Li, Yong Cao, Ya Gao, Wei Zhang

**Affiliations:** 1 Marine College, Shandong University at Weihai, Weihai, 264209 China; 2 State Key Laboratory of Dao-di Herbs, National Resource Center for Chinese Materia Medica, China Academy of Chinese Medical Sciences, Beijing, 100700 China; 3 School of Basic Medical Sciences, Shandong University, Jinan, 250012 China

## Abstract

Although molecular data have revealed huge amounts of plant diversity, interpreting genetic diversity into entities corresponding to species is still challenging. Taxonomic ranking based on genetic distance has been used extensively, but the results have been open to dispute, while the application of the strategy to plants has been restricted to a small number of cases. Here, levels of internal transcribed spacer 2 (ITS2) sequence variation were examined from 17,203 sequences, representing 5,439 species in 113 genera of seed plants, to ascertain the association between species status and their molecular divergence. Our results showed that, although the average genetic distances of sister species (AGDS) varied among angiosperms, the mean value was 3.98% and seemed not to be influenced by higher-level hierarchical classification or life history. AGDS was also stable within the major lineages of the gymnosperms but at approximately half the value of angiosperms, except for the Gnetidae, where the AGDS almost equaled that of angiosperms. We found that these AGDS discrepancies, associated with the rates of molecular evolution, cannot simply be attributed to generation-time differences, and highlight the complex life histories of plants. Our results provide general ITS2 thresholds in seed plants, and suggest their use in species identification.

## Introduction

Accurate species delimitation and identification are the prerequisites for many areas of biology, including ecology, evolution and conservation^1^. The interest in this issue over the past two and a half centuries has led to no fewer than 24 species concepts and their associated operational criteria^2^. Currently, the phylogenetic species concept (PSC) dominates plant taxonomy^3^. However, applying PSC has been found to greatly increase the number of species; for example, Apagow showed that, using PSC, the number of species grew by 121% compared with that determined by non-PSC methods in his review of 91 case studies across a broad range of biological groups^4^. Similarly, the current popular technique of DNA barcoding has also resulted in an unwarranted explosion of species numbers, mainly through the splitting of existing species, rather than the identification of new species^5^. Theoretically, even the tiniest subdivision of a species can be made diagnosable if the molecular markers have adequate discriminatory power. Overall, in the absence of other information, the currently widely-used PSC or DNA barcoding methods are still inadequate for species delimitation or identification.

“Threshold of genetic distance” has been suggested as a solution to avoid false-positive detection in tree-based methods^6–9^. It can even be used independently of tree shapes for unknown assignments, when a prior threshold value has been established, based on the use of sufficient samples of closely-related taxa, and specimens yielding sequences below that threshold value will be considered conspecific^9^. More generally, some threshold values of genetic divergence have gained broad application to help species discrimination, notably the “97% rule” of 16S rDNA in microbial life^10^, the 2% mitochondrial cytochrome c oxidase (COI) threshold or the “10× rule” in animals^9,11^. It must be admitted that the use of threshold values is not without its problems. For example, no fixed threshold of COI can apply to all taxa because of variation in intraspecific divergence, or discrepancies between genetic divergence and taxonomic assignment because of introgression, incomplete lineage sorting or recent speciation^12–14^. However, a relative threshold value remains a useful screening tool to help build a species partition hypothesis where groups have not received intensive taxonomic analysis^8,11^.

Our current knowledge of genetic distance and the debates concerning thresholds for taxonomic assignments have been derived largely from comparative studies of the mitochondrion-encoded gene COI of animals^8,9,11–14^. It is surprising that such studies are scarce in plants and it remains unclear what extent and correlates of genetic divergence exist among plant species. Like COI in animals, the internal transcribed spacer region (ITS/ITS2) of rDNA has been widely used, and has contributed greatly to plant taxonomy, and extensive ITS/ITS2 sequences across a broad range of plant diversity are available in GenBank. More importantly, the ITS region is not expressed; it is removed via splicing during transcript processing, and thus would be subject to lower functional constraints than COI, offering a larger number of nucleotide sites that could evolve in an essentially neutral way^15^.

This study presents the ITS2 sequence divergence between sister species (closely-related species, being descendants of the same ancestral species) across a broad range of plant groups, with the general aims of testing the extent and association between ITS2 divergence and plant species boundaries, and, if possible, to define a molecular threshold to facilitate taxonomic decisions. Specially, we report for the first time the variation in ITS2 divergence between sister species among the main groups of seed plants, and provide some novel insights into the molecular evolutionary rate in seed plants.

## Results

### Sequences and taxon analyses

A total of 32,648 ITS2 sequences were obtained from GenBank, including 7,866 species, 179 genera, 99 families and 54 orders (Supplementary Table S1), which covered the majority of angiosperm (Supplementary Fig. 1) and gymnosperm lineages except for the monotypic Ginkgoidae. Only those genera for which sister species were available were considered for further analysis, and finally 17,203 sequences, involving 5,439 species and 113 genera, were used for analysis (Supplementary Table S2). Of these, 237 species (4.26%) were definitely paraphyletic or polyphyletic, and only 570 species (10.24%) were unambiguously identified as monophyletic, due mainly to many others lacking adequate species resolution in neighbor-joining (NJ) trees. As a result, 281 sister-species pairs were extracted, including 251 angiosperm species and 30 gymnosperm species (Supplementary Table S3). In addition, 10,417 *rbcL* sequences (a plastid gene encoding the large subunit of ribulose-1,5-biphosphate carboxylase/oxygenase) were obtained from plants, including 2,289 species, 102 genera, 40 families and 30 orders, and these were analyzed and compared with those of ITS2 (Supplementary Table S4).

### Comparison of sequence variation between *rbcL* and ITS2

**Figure 1:**
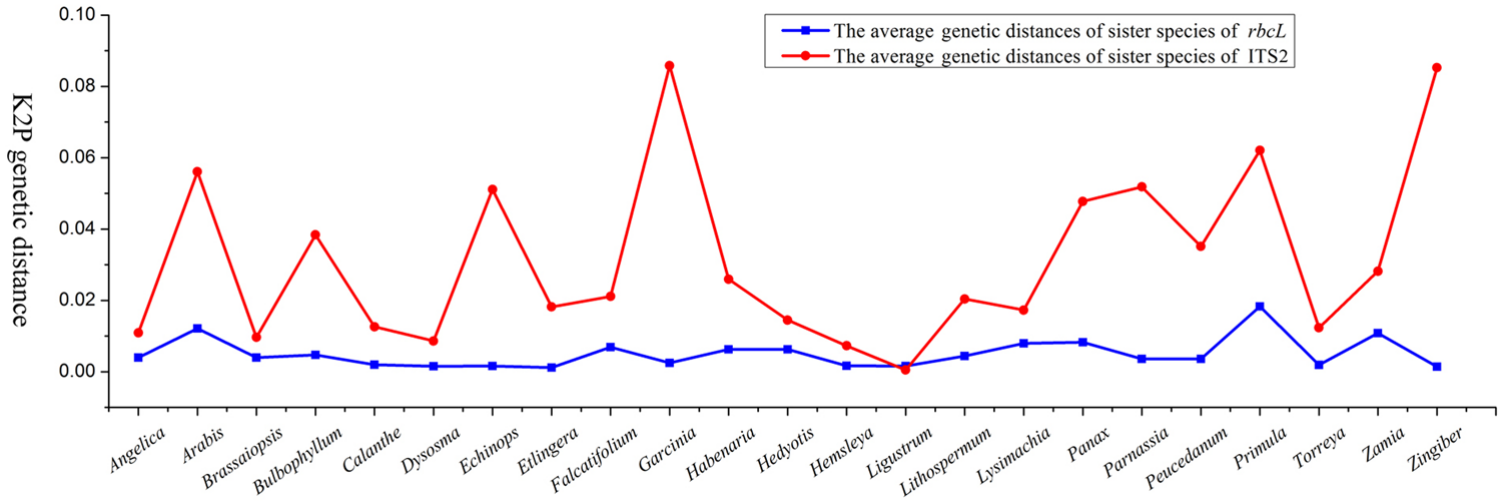
Comparison of the average genetic distances of sister species between *rbcL* and ITS2.

We compared the average genetic distances of sister species (AGDS) between *rbcL* and ITS2 within the common 23 genera, and found that the mean *rbcL* AGDS (0.51%) was only about one-sixth that of ITS2 (3.14%) (Supplementary Table S5). In addition, the sequence variation showed significant difference between the two DNA regions (*P* = 0.00) (Table 1, Fig. 1). Given the extremely low evolutionary rate of the *rbcL* sequence, utilization of variation in this vital plant gene could not distinguish the most closely-related species, especially sister species. We thus focused on the more variable ITS2 region instead.

**Table 1:** Nonparametric test of ITS2 AGDS variations from comparative datasets.

Comparisons	*P* value	Test results
ITS2 vs. *rbcL*	0.00	significant differences
angiosperm vs. gymnosperm	0.003	significant differences
(Pinidae + Cycadidae) vs. Gnetidae	0.676	not significant
angiosperm vs. Gnetidae	0.268	not significant
angiosperm vs. (Pinidae + Cycadidae)	0.006	significant differences
monocotyledons vs. dicotyledons	0.786	not significant
herbaceous plants vs. woody plants	0.086	not significant

### ITS2 sequence variation at the sister species, inter- and intraspecific taxonomic levels

**Figure 2:**
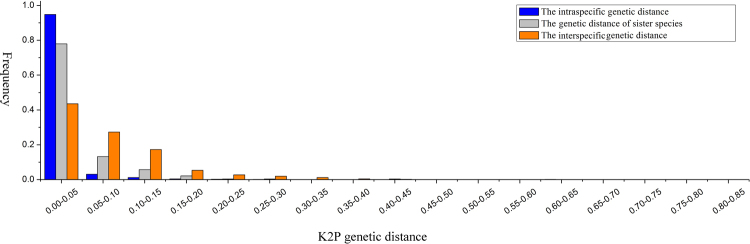
Frequency distribution of three types of ITS2 molecular divergence.

ITS2 genetic distances between sister-species, inter- and intraspecific taxa within each genus were analyzed, and the results showed that they varied over a wide range among all the seed plants investigated, but were mainly concentrated within a certain range (Fig. 2). The interspecific genetic distance, ranging from 0 to 84.5%, was the most variable parameter, followed by sister-species and intraspecific values. The AGDS in seed plants (3.76%) was more than three times the intraspecific genetic distance and about half the interspecific genetic distance value.

### Comparison of the average genetic distances of sister species among different groups

**Figure 3:**
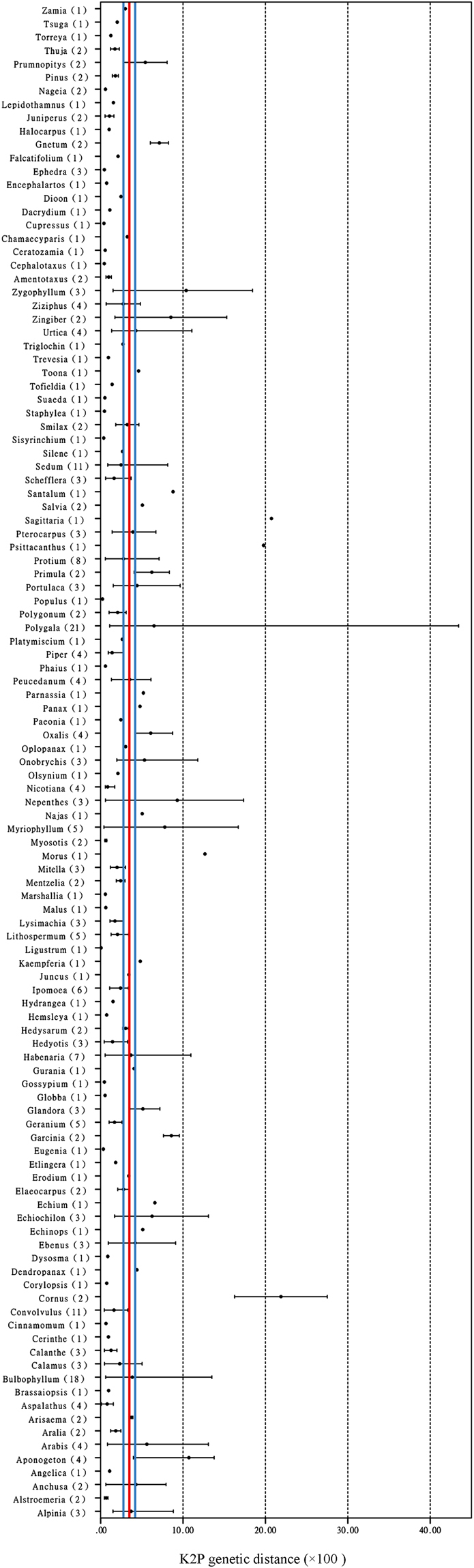
Comparisons of ITS2 genetic distance of 281 sister-species pairs in seed plants. Average genetic distances are shown as dots; bars represent the range of values obtained within a sister species pair, and sample sizes are shown in parentheses. The red line indicates the average genetic distances of 281 sister species, and the range between the blue lines represents the 95% confidence interval.

We analyzed the average genetic distances of 281 sister-species pairs in seed plants and found that they varied at different taxonomic levels but still showed some stability in certain groups (Fig. 3). Among all the species within the seed plants, the AGDS values ranged from 0.05% (*Ligustrum micranthum* vs. *L. japonicum*) to 43.45% (*Polygala furcata* vs. *P. tatarinowii*) (max/min = 851.92), but fluctuated relatively little within a genus (max/min = 41.71 in *Myriophyllum*) (Supplementary Table S6). Although the distance values varied greatly in a few extreme cases, 90% of the values were concentrated within the range 0–10.00%, with the mean value of 3.76% (95% confidence interval, CI: 3.20–4.32%) (Figs 2 and 3).

Interestingly, we found that the mean AGDS in angiosperms (3.98%) was 2.04 times that in gymnosperms (1.95%); as a consequence, we analyzed them separately (Table 2). In gymnosperms, although the AGDS ranged from 0.40% (*Cupressus tonkinensis* vs. *C. chengiana*) to 8.23% (*Gnetum latifolium* vs. *G. neglectum*) (max/min = 20.58) in a small number of cases, the mean values were relatively stable. For example, the AGDS in the Pinidae and Cycadidae were very similar to one another (1.73% and 1.64%, respectively). An exception was the Gnetidae, with its AGDS being similar to that of the angiosperms (3.12% vs. 3.98%) (Tables 1 and 2).

**Table 2:** Comparison of ITS2 genetic distance among different groups of seed plants.

Taxon	Genetic distance	Average value	95% Confidence interval	Variation		N
Spermatophyte	Intraspecific	1.19%	[1.06%, 1.32%]	[0, 64.00%]		2,476
	Sister species	3.76%	[3.20%, 4.32%]	[0.05%, 43.45%]		281
	Interspecific	7.77%	[7.75%, 7.80%]	[0, 84.53%]		293,277
Angiosperm	Intraspecific	1.20%	[1.06%, 1.34%]	[0, 64.00%]		2,247
	Sister species	3.98%	[3.36%, 4.59%]	[0.05%, 43.45%]		251
	Interspecific	7.88%	[7.85%, 7.91%]	[0, 84.53%]		285,837
Monocotyledon	Intraspecific	1.51%	[1.15%, 1.88%]	[0, 64.00%]		626
	Sister species	3.90%	[2.78%, 5.02%]	[0.37%, 20.73%]		62
	Interspecific	8.55%	[8.50%, 8.61%]	[0, 84.53%]		77,533
Dicotyledon	Intraspecific	1.08%	[0.95%, 1.21%]	[0, 43.19%]		1,621
	Sister species	4.00%	[3.27%, 4.73%]	[0.05%, 43.45%]		189
	Interspecific	7.69%	[7.66%, 7.71%]	[0, 69.07%]		226,832
Gymnosperm	Intraspecific	1.10%	[0.77%, 1.44%]	[0, 22.63%]		229
	Sister species	1.95%	[1.18%, 2.72%]	[0.40%, 8.23%]		30
	Interspecific	3.76%	[3.65%, 3.87%]	[0, 60.65%]		7,440
Pinidae	Intraspecific	1.00%	[0.55%, 1.44%]	[0, 19.07%]		113
	Sister species	1.73%	[0.97%, 2.48%]	[0.40%, 8.07%]		21
	Interspecific	4.87%	[4.72%, 5.02%]	[0, 20.98%]		3,343
Cycadidae	Intraspecific	0.98%	[0.32%, 1.63%]	[0, 22.63%]		79
	Sister species	1.64%	—	[0.55%, 2.82%]		4
	Interspecific	2.10%	[2.02%, 2.18%]	[0, 15.24%]		2,896
Gnetidae	Intraspecific	1.68%	[0.94%, 2.42%]	[0, 9.85%]		37
	Sister species	3.12%	—	[0.45%, 8.23%]		5
	Interspecific	4.68%	[4.22%, 5.14%]	[0, 60.65%]		1,201

In angiosperms, the mean AGDS (3.98%, 95% CI: 3.36–4.59%) was relatively stable in comparable groups: for example, the monocotyledons (3.90%, 95% CI: 2.78–5.02%) vs. the dicotyledons (4.00%, 95% CI: 3.27–4.73%), and herbaceous plants (4.10%, CI: 3.37–4.82%) vs. woody plants (3.23%, CI: 2.33–4.22%) (Table 1). Furthermore, we selected three distinct lineages relatively rich with respect to sister species, according to the Angiosperm Phylogeny Group (APG), namely the Asparagales (monocots), Fabales (rosids) and Apiales (asterids). The mean AGDS values of these lineages were 3.28% (95% CI: 2.02–4.54%), 5.08% (95% CI: 2.59–7.57%) and 2.52% (95% CI: 1.55–3.49%), respectively, and their overall mean value (3.13%) approximated to that of the angiosperms (Supplementary Table S7).

### Test of discriminatory powder of AGDS method from a single plant system

**Figure 4:**
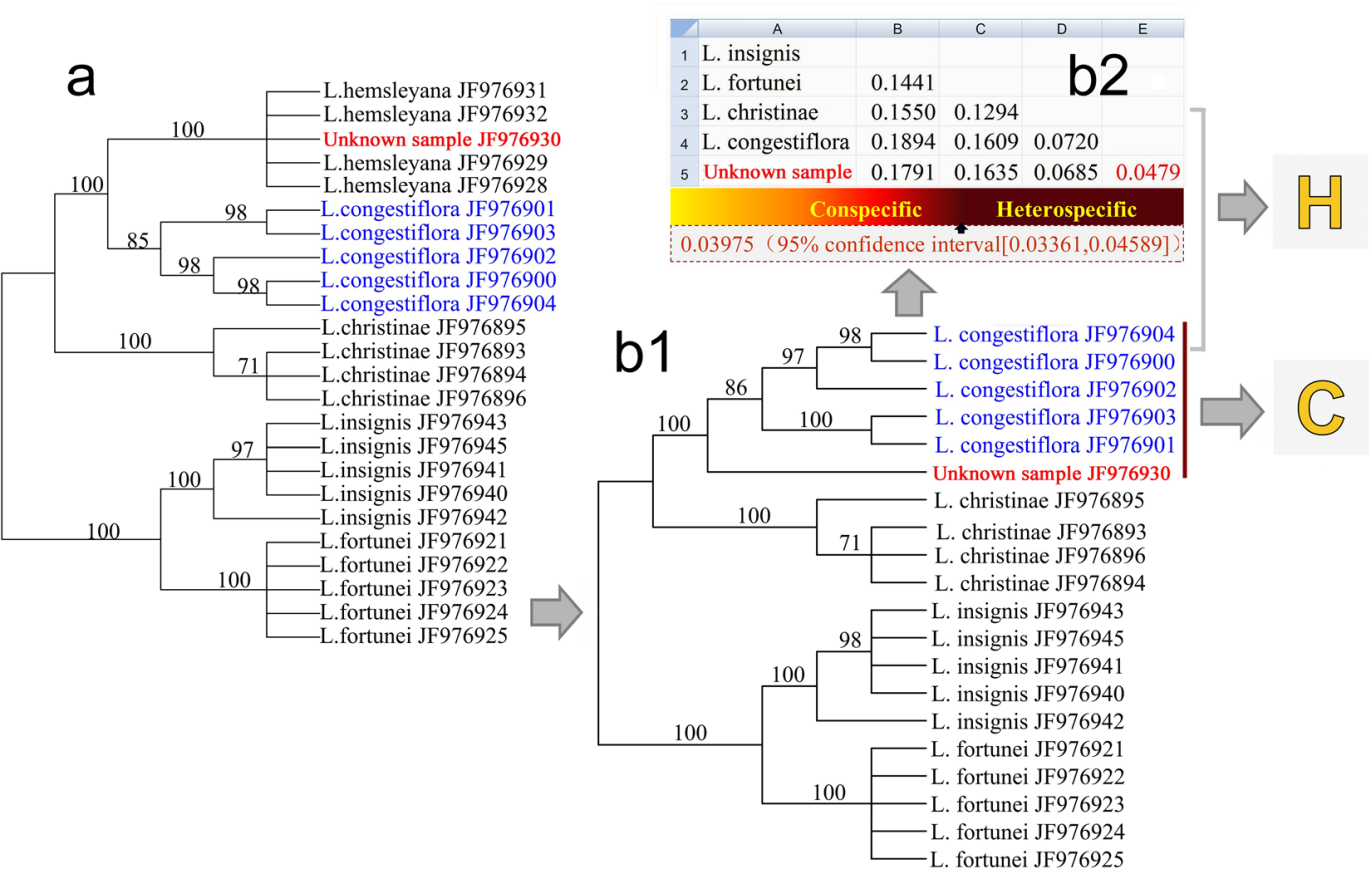
A schematic diagram illustrating how the ITS2 sequence-divergence threshold improves species identification accuracy when using DNA barcoding. (**a**). An unknown sample is successfully assigned into its conspecific clade when the DNA barcoding library was complete. (**b1**). The unknown sample is assigned into an alternative clade when the DNA barcoding library was incomplete and could easily be mistakenly identified. (**b2**). K2P genetic distances between species are calculated from b1. The sequence-divergence values between the unknown sample and *L. congestiflora* (4.79%) is above the ITS2 AGDS threshold (3.98%), indicating that they are heterospecific.

We selected *Nicotiana* (Solanaceae), a more species-diversified genus, to test the species discriminatory power of the AGDS method in a real plant system. In this study we sampled 67 species, representing 87% species coverage of this genus. The calculation of the average genetic distance between species in this sample generated 2,211 pairwise values, of which 1,789 pairwise values were greater than the 3.98% threshold (Supplementary Dataset 1). This result indicated that 80.91% species of *Nicotiana* could be identified through the AGDS method. In contrast, DNA barcoding analysis of these species yielded a tree of mostly polytomies, from which only five species (7.46%) were clustered as a monophyletic group and thus could be identified (Supplementary Fig. S2). Furthermore, we chose some closely-related species of *Lysimachia* (Primulaceae) to test the advantage of the AGDS method when DNA barcoding library was incomplete. Species *L. hemsleyana* and *L. congestiflora* were most closely related and they clustered together as sister species in our NJ tree. An unknown sample was successfully identified as *L. hemsleyana* from DNA barcoding because it nested within the *L. hemsleyana* clade with 100% support value (Fig. 4a). However, when the reference sequences of *L. hemsleyana* were not represented in the DNA barcoding library, the unknown sample and *L. congestiflora* individuals were clustered together into a 100% supported clade and thus could easily be mistaken for *L. congestiflora* (Fig. 4 b 1). We calculated the genetic distance between the unknown sample and *L*. *congestiflora* individuals and found that its value (4.79%) were greater than the 3.98% threshold, indicating that the unknown sample and *L. congestiflora* were heterospecific.

## Discussion

### Evolutionary significance of genetic distance between sister species

ITS2 has been suggested as one of the standard core barcodes and also the only nuclear barcode available for the identification of seed plants^16–18^. However, there is still debate over its merits in plant identification, mainly due to lack of comprehensive and multi-taxon testing. Our sister-species comparisons to identify a divergence threshold value across a broad phylogenetic diversity range could offer a statistically-rigorous approach for comparing evolutionary rates, as emphasized by Barraclough^19^. In this study, we compared the AGDS between ITS2 and *rbcL*, the two core barcode sequences suggested for use in plant identification, and found that the variation in ITS2 sequences was six times greater than that of *rbcL*. This result was consistent with the previous broad surveys of base substitution rates between nuclear and chloroplast genes^20^, and confirmed the validity of our sister-species-based method. One key finding here was that the average rate of ITS2 genetic change in herbaceous plants was 1.3 times higher than that for woody plants (*P* = 0.086) within the angiosperms. This result agrees with that of Soria-Hernanz *et al*.^21^, who showed that annual plants evolved at a rate on average 1.4–1.6 times faster than that of perennials, based on ITS sequence. It is noteworthy that our current knowledge of the association between molecular evolution rates and plant life history has been derived largely from comparative studies on angiosperms. Until recently, few studies had been carried out on gymnosperms. Notably, we found that the average ITS2 genetic change per species in angiosperms was 2.1 times that in gymnosperms (*P* = 0.003). The rate in the Pinidae was similar to that in the Cycadidae within the gymnosperms but was about one half that in the Gnetidae, and it was interesting to note that the AGDS of the Gnetidae was closest to that of the angiosperms.

Taken together, these observations suggest that, because the range of life histories in plants is more complex than that in animals, the differences in molecular evolution rate among plants cannot simply be interpreted using the generation time theory as advocated for animals by Whittle & Johnston^22^. It has been reported that the haploid stage of angiosperms, whether woody or herbaceous, is equally short, while that of gymnosperms can be extremely long, with the exception of the Gnetidae. The time taken from pollination to fertilization in pine, for example, is about one year, in ginkgo is about four to five months, but in ephedra (Gnetidae) is about 10 to 30 hours^23^, similar to the value in angiosperms. Thus, the haploid stage duration could better explain why the herbaceous and woody plants of the angiosperms, together with the Gnetidae, have equally fast ITS evolutionary rates, contrasting significantly with those of the Pinidae and the Cycadidae in the gymnosperms. In summary, our alternative analyses of molecular evolution rates, based on the sister-species divergence, have some biological support and provide new insights into plant molecular evolution.

### The threshold value of ITS2 and its significance in future species delimitation and identification

Our research argues that the current widely-used mean congeneric genetic distance overestimates interspecific variation and may lead to misidentification^24^. This view was confirmed here by our use of sister-species distances and found that their average value was only one-half of the interspecific (congeneric) value. In general, the average genetic distances between sister species was about three times higher than that of intraspecifics, in contrast with the 10× rule in animals^11^.

Another striking empirical finding noted here is that the AGDS were relatively constant and are probably not influenced by taxonomic category or life history. These findings may suggest universal species molecular thresholds that could be used to help species delimitation and identification. Although DNA barcoding is currently the most popular method for species identification, it should be used with caution because most taxon reference libraries are still largely incomplete, except for a few well-known and well-studied taxonomic groups^9^. In operation, whenever a query (unknown sample) lacks a conspecific target, it will either be erroneously assigned to the most similar heterospecific sequence existing in the database, or misidentified as a novel species. The seriousness of the problem can be reduced, however, by setting a divergence threshold value, above which the query is discarded (Fig. 4). In practice, people using molecular barcoding usually want to determine whether an unknown sample was a particular species in which they are interested, rather than to identify the species if it is not their species-of-interest. For example, although there are more than 1,400 *Solanum* species in the world, quarantine staff in China are concerned only with four invasive species, namely *S. carolinense*, *S. elaeagnifolium*, *S. rostratum* and *S. torvum* ^25^. Under such conditions, it is only necessary to compare the sequence of the query with sequences of the four target species, using a threshold value; the greater they diverge, the less likely that they are conspecific.

Traditionally, species are mainly identified as the smallest monophyletic group according to the phylogenetic species definition in current DNA taxonomy. Some authors worry that the mounting use of DNA taxonomy could lead to an increase in numbers of species and cause problems of taxonomic inflation^5^. In this case, it is helpful to provide a divergence threshold as a reference point for determining a species boundary, below which a query will be considered conspecific. It is noteworthy that genetic analysis of a single locus is not robust enough to define a reliable species boundary, and may be limited by some evolutionary scenarios, such as adaptive radiation, hybridization or introgression. Thus, one should keep in mind that our divergence threshold is not indicative of final species delimitation but merely a primary species hypothesis, and that further testing, using additional data, such as multi-locus, morphological or ecological ones, are essential to proposing reliable species hypotheses.

## Materials and methods

### Taxon sampling and sequence acquisition

Taxa were retrieved from the literature on plant DNA barcodes through the Web of Science Service, mainly because these taxa most possibly have solid foundations for their species boundaries as a result of integrative taxonomy. Most importantly, they have adequate inter- and intraspecific sampling to meet DNA barcode requirements. For the various groups of seed plants, all entries with the annotation “internal transcribed spacer 2” or “ITS2”, corresponding to these taxa, were retrieved from GenBank. We referred to the APG classification to ensure that our sampling could cover as much of the diversity of flowering plants as possible^26,27^. In addition, we examined species coverage and validity of species names according to the web server *The Plant List* (http://www.theplantlist.org/).

### Phylogenetic analyses

The ITS2 region was delimited based on the GenBank annotation or the ITS2 database web server (http://its2.bioapps.biozentrum.uni-wuerzburg.de.). The sequences of each genus were separately aligned with Clustal X^28^ and adjusted manually using BioEdit 7.0.5.^29^ The NJ tree recommended as the standard barcoding method was adopted and performed with MEGA 6.0^30^ based on the Kimura 2-parameter (K2P) model, using 1000 replicates to estimate the confidence of the clades. Sister-species pairs were identified according to the tree topology, together with the criteria of reciprocal monophyly^6^.

### Molecular divergence

The sequence divergence of ITS2 between sister species was measured as a species-level genetic difference, and calculated using the K2P distance model through MEGA 6.0. For comparison, inter- and intraspecific variation in the ITS2 sequence within each genus were also calculated. To explore sequence variation patterns and their possible mechanisms, we analyzed three molecular divergences in higher taxonomic groups, namely gymnosperms vs. angiosperms, herbaceous plants vs. woody plants, and dicotyledons vs. monocotyledons, using SPSS 22.0 (SPSS, Chicago, IL, USA), and presented with OriginPro 9.0 (Originlab Corporation, Northampton, MA, USA).

## Electronic supplementary material

Supplementary Table S1

Supplementary Table S2

Supplementary Table S3

Supplementary Table S4

Supplementary Table S5

Supplementary Table S6

Dataset 1

Supplementary materials for Figure S1 Figure S2 and Table S7
