# Supplementary material for: Molecular thresholds of ITS2 and their implications for molecular evolution and species identification in seed plants
Source: Sci Rep. 2017 Dec 11;7:17316. doi: 10.1038/s41598-017-17695-2 (PMC5725418; doi:10.1038/s41598-017-17695-2)
Supplement: Supplementary file 8 — Supplementary materials for Figure S1 Figure S2 and Table S7 [file 41598_2017_17695_MOESM8_ESM.pdf]

Supplementary Information

Molecular thresholds of ITS2 and their implications for molecular evolution and species identification in seed plants

Ying Qin[1,3], Meihui Li[1], Yong Cao[1], Ya Gao[1], Wei Zhang[1,2]

1. Marine College, Shandong University at Weihai, Weihai, 264209, China
2. State Key Laboratory of Dao-di Herbs, National Resource Center for Chinese Materia Medica, China Academy of Chinese Medical Sciences, Beijing 100700, China
3. School of Basic Medical Sciences, Shandong University, Jinan, 250012, China

Ying Qin and Meihui Li contributed equally to this work. Correspondence and requests for materials should be addressed to Wei Zhang (email:wzhang@sdu.edu.cn)

This file includes

Supplementary Figure 1

Supplementary Figure 2

Supplementary Table 7

(Supplementary Tables 1-6 and Dataset 1 are large datasets and included as separate Excel documents)

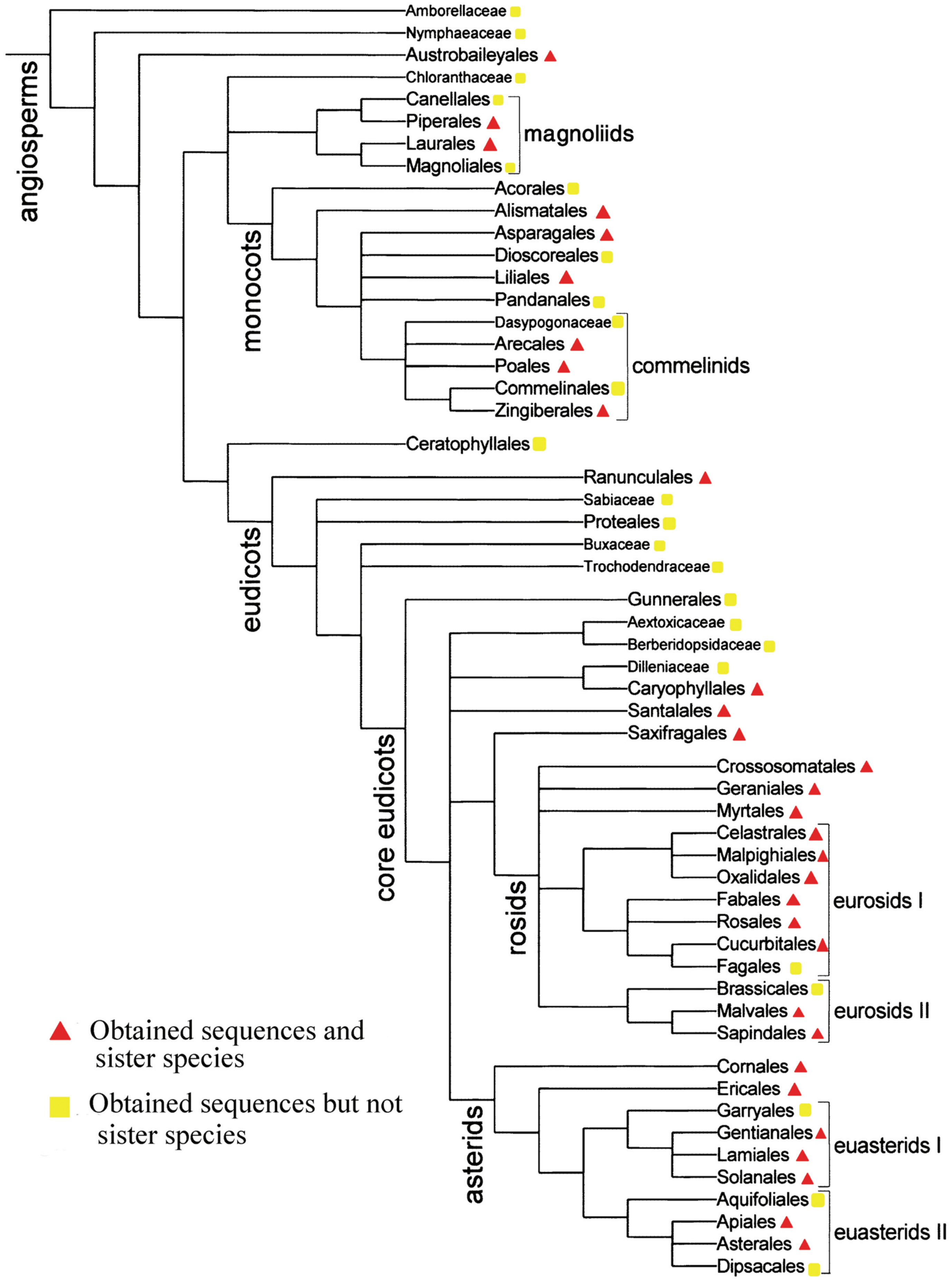

Supplementary Figure S1. The phylogenetic position of investigated taxa in the APG framework

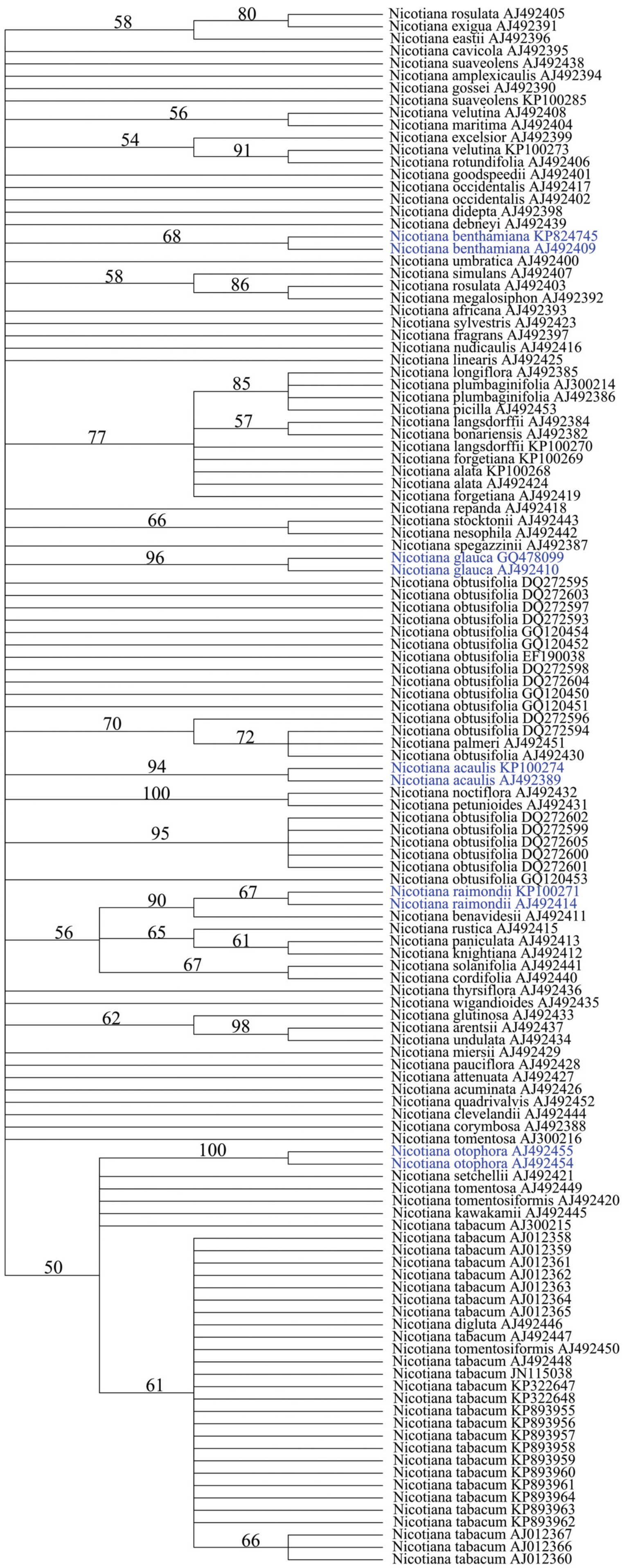

Figure S2. A neighbor joining tree of *Nicotiana* plants based on ITS2 sequence. Bootstrap values (＞50%) are shown above the branches. Numbers followed taxon names are GenBank accession numbers. Successfully identified species are highlighted in blue.

Supplementary Table S7. Comparison of ITS2 genetic distance for the selected groups of species-rich orders

Taxon	Average value	95% Confidence interval	Variation	N
Asparagales	3.28%	[2.02%,4.54%]	[0.37%,13.49%]	31
Fabales	5.08%	[2.59%,7.57%]	[0.09%,43.45%]	37
Apiales	2.52%	[1.55%,3.49%]	[0.60%,6.10%]	15
Pinales	1.65%	[0.96%,2.33%]	[0.40%,3.23%]	9
All	3.13%	[2.63%,4.82%]	[0.09%,43.45%]	92
